# Photobiomodulation therapy in oral mucositis prevention and treatment: insights from a bibliometric review

**DOI:** 10.1007/s00520-026-10976-5

**Published:** 2026-07-16

**Authors:** Camila Maria Silva, Izabella Maria Barboza da Silva, José Ricardo Dias Pereira, Ana Carolina Prado Ribeiro-Silva, Luiz Alcino Gueiros

**Affiliations:** 1 Graduate Program in Dentistry, Science Health Center, Universidade Federal de Pernambuco, Cidade Universitária, Av. Prof. Moraes Rego, 1235, CEP: 50.670-901 Recife, PE Brazil; 2 Dental Oncology Unit, Instituto Do Câncer Do Estado de São Paulo Octavio Frias de Oliveira (ICESP), São Paulo, Brazil; 3 Oral Medicine Unit. Hospital das Clínicas – EBSERH, Universidade Federal de Pernambuco, Recife, Brazil

**Keywords:** Oral mucositis, Cancer, Chemotherapy, Radiotherapy, Low level laser therapy, Photobiomodulation

## Abstract

**Purpose:**

This study presents the first bibliometric analysis aimed at evaluating the current state of knowledge and identifying limitations regarding the use of photobiomodulation (PBM) in the prevention and treatment of oral mucositis.

**Methods:**

A comprehensive search of the SCOPUS database was undertaken, with bibliometric analyses conducted using the Bibliometrix R-tool, Microsoft Excel, and VOSviewer.

**Results:**

A total of 725 articles were retrieved, of which 526 fulfilled the eligibility criteria. The 100 most cited articles, published between 1993 and 2025, were selected for analysis. The list comprised 56 primary studies and 44 secondary studies, collectively authored by 610 individuals, yielding an average of 9 authors per article. Research was predominantly concentrating studies focused on the efficacy of photobiomodulation in treating oral mucositis. The USA and Brazil emerged as leading contributors for the highest number of citations and scientific publications, both independently and in international collaborations. Among scientific journals, *Supportive Care in Cancer* stood out, with 14 of the most cited documents on the subject.

**Conclusion:**

The list of articles included in this review serves as a relevant source of quantitative data, highlighting studies considered as references, as well as authors, journals, and countries with the highest scientific output. Additionally, it provides emerging insights on the topic.

**Supplementary Information:**

The online version contains supplementary material available at 10.1007/s00520-026-10976-5.

## Introduction

Oral mucositis (OM) is a frequent adverse event during cancer treatment secondary to radiotherapy (RT), high-dose chemotherapy (CT), a combination of RT and CT (RT-CT), and hematopoietic stem cell transplantation (HSCT), characterized by erythema and ulceration of the oral mucosa and gastrointestinal tract [1]. The incidence rate of oral mucositis varies considerably, being observed in around 40% of patients undergoing chemotherapy. However, this percentage increases in patients with head and neck cancer (HNC) treated with both chemotherapy and radiotherapy, with an occurrence of approximately 90% [2]. Similarly, between 60 and 80% of patients receiving high-dose chemotherapy as conditioning for hematopoietic stem cell transplantation will develop OM [3].

OM may be associated with intense pain, increased consumption of opioids (narcotics), eating difficulties, increased need for parenteral nutrition, increased risk of bacteremia, and decreased quality of life [4]. Despite the wide variety of proposed treatments, only a few have proven to be truly effective. Suggested interventions for symptomatic control include povidone-based mouthwashes, antibiotics, anti-inflammatory agents, analgesics, topical anesthetics, and calcium phosphate rinses, among other alternatives [5]. However, photobiomodulation (PBM) is the treatment with the most evidence of efficacy in OM prevention [4].

Although several studies demonstrate the efficacy of this therapy in both treatment and prophylactic effects, the results may be influenced by the different protocols adopted by each unit [6]. The Mucositis Study Group of the Multinational Association of Supportive Care in Cancer/International Society of Oral Oncology (MASCC/ISOO) published evidence-based clinical guidelines for OM, including a section about laser and light therapy, and recommends a therapeutic dose of 4 J/cm^2^ [4]. The parameters to be observed include the following: intensity (power, mW), energy density (mW/cm^2^), energy (J), treated area size (cm^2^), time (seconds), number of points treated, distance from the device or contact with tissue, mode of operation, and the duration of oncological treatment [4]. Currently, it is recommended to use low-power lasers with wavelengths between 640 and 940 nm, and that this application be performed locally on the lesion. Low-intensity lasers increase cellular metabolism, stimulate mitochondrial activity, and act as analgesics, anti-inflammatories, and mucosal injury repairers [6].

PBM is undeniably a significant advancement in OM treatment. In this perspective, bibliometric review allows for an evaluation of the most cited studies on the topic, enabling the establishment of the academic influence of each article in its specific field and identifying new research trends in the area, confronting them with the existing scientific framework regarding the use of photobiomodulation in oral mucositis after anticancer treatment. Therefore, the aim of this article was to analyze the current knowledge and its limitations regarding the use of photobiomodulation in the prevention and treatment of OM, through a bibliometric review of the 100 most cited articles on the topic.

## Methodology

This study is a bibliometric review, in which a systematic quantification of the existing scientific bibliographic material was conducted, using statistical and mathematical methods to establish what is called bibliometrics. This study does not require approval from an ethics committee, as the data used are in the public domain. ChatGPT v5.0 was used for language editing and style checking.

A search was conducted using the SCOPUS database in April 2024, through the search string (“mucositis” OR “oral mucositis”) AND (“laser” OR “lasertherapy” OR “photobiomodulation” OR “low level laser” OR “photobiomodulation therapy”). The records found were listed in ascending order, starting from the most cited for selection. The selection was made through the reading of their abstracts by two independent evaluators; any disagreements were discussed and resolved by consensus between the evaluators, and if consensus was not reached, a third evaluator was consulted to break the tie. The first 100 most cited studies were selected to be part of this study, with no specific time frame for the publications. Articles not written in English or Portuguese, studies that deviated from the proposed topic, and texts that were not available in full for reading were excluded.

Only original articles were considered, excluding, for example, letters to the editor. No duplicates were found. Through this methodology, the following information was compiled: full authorship, publication title, year of publication, number of citations, citation density, journal and its impact factor, institution and country of the first and last authors, number of authors, number of centers, number of countries, collaboration index, most productive authors, and author bibliometric indices (H-index, G-index, M-index).

Data analysis was performed using descriptive statistics, expressed in median values or percentages calculated in Excel software (Microsoft Inc., Redmond, WA, USA). An R software program [7] with the Bibliometrix package [8], an open-source tool for comprehensive analysis and mapping of scientific literature, was used to retrieve all lists related to authors, countries, and their interactions; additionally, it was used to generate statistical charts and scientific maps. Furthermore, VOSviewer version 1.6.17 [9], a software for bibliometric mapping, was also used. These programs were used to generate scientific maps, complementing the information from Bibliometrix.

## Results

### Articles

Through the developed search key, 725 studies were located in the SCOPUS database. No duplicates were found. Of these, 199 were excluded, and 526 were included for analysis. The 100 most cited articles were selected, published between 1993 and 2022, and with citation counts ranging from 868 to 43, as shown as online resource 1. Thirty-one articles are considered classic articles (with over 100 citations).

### Annual distribution

When analyzing the annual distribution of scientific output over the years among the articles included in the analysis, stability was observed in the number of articles during the 1990 s to the early 2000 s, in contrast to the growing concentration of studies in the subsequent decades, reaching the highest concentration of published articles in 2016 (*n* = 9) among the most cited, as shown as online resource 2.

### Type of study

Among the included articles, 56% were primary studies, and 44% were secondary studies. The primary and secondary studies were distributed, as shown in Table 1. A higher concentration of clinical trials was observed (*n* = 41) with a total of 2499 participants included (61 patients/study), followed by narrative reviews (*n* = 20) and systematic reviews (*n* = 17).

**Table 1:** Distribution of primary and secondary studies among the most cited papers

Study	Type of study	Distribution
Primary (*n* = 56)	Case report	1 case report among primary studies
	Case-control Studies	3 case–control
	Preclinical Studies	6 animal studies and 5 cell studies
	Clinical Trials	41 clinical trials, including non-randomized (*n* = 9), single-blinded (*n* = 13), double-blind (*n* = 16), and triple-blind (n = 03) studies
Secondary (*n* = 44)	Systematic Reviews	17 systematic reviews summarize findings across multiple studies
	Review	20 narrative review
	Guidelines	7 guidelines providing recommendations evidence-based clinical practice

**Fig. 1:**
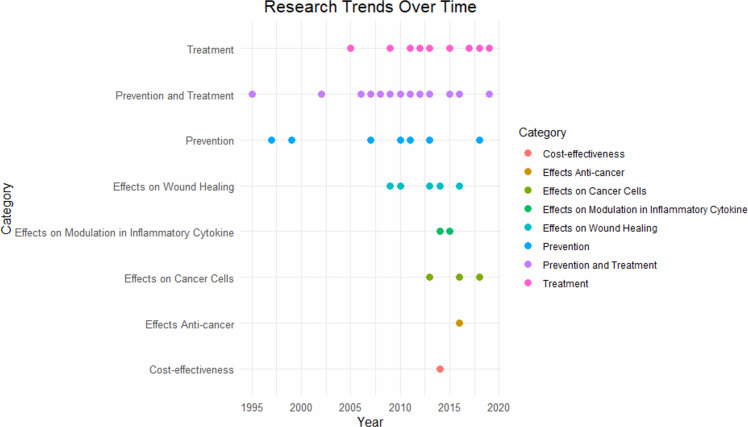
Research trends topics over time

Over time, the most cited primary studies have generally focused on the use of laser therapy for the prevention and treatment of oral mucositis, particularly analyzing its concomitant effects. It is worth highlighting that, among the clinical trials, the main research topic was the use of photobiomodulation in the treatment and prevention of oral mucositis, with approximately 42% (*n* = 17) of the studies addressing treatment, 39% (*n* = 16) both prevention and treatment, and 19% (*n* = 8) prevention only. Few studies were related to the effects on cancer cells and the modulatory mechanisms of laser therapy on inflammatory cells and effects on wound healing (Fig. 1).

### Keywords

**Fig. 2:**
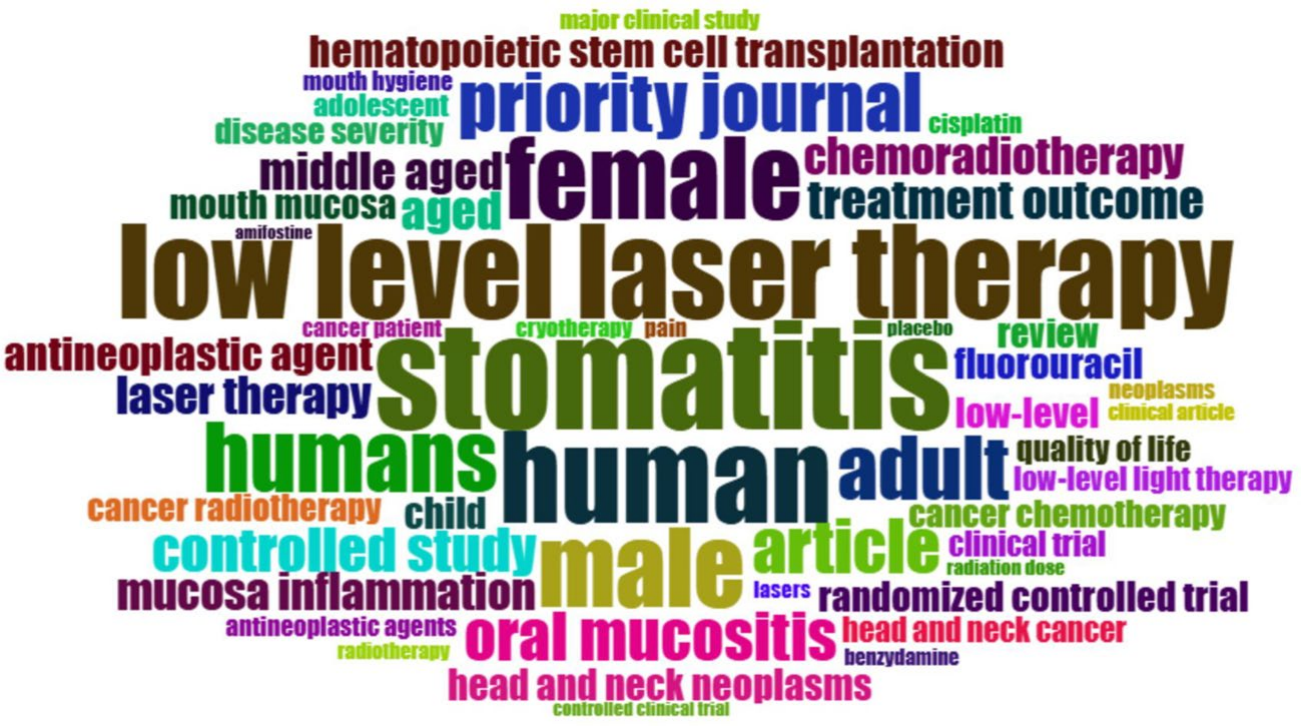
Keywords cloud

The image shows the most frequently used keywords among the authors in the 100 most cited articles, with prominent terms such as low-level laser therapy, stomatitis, oral mucositis, mucosa inflammation, and head and neck neoplasm, as shown in Fig. 2.

### Author and co-authorship

**Fig. 3:**
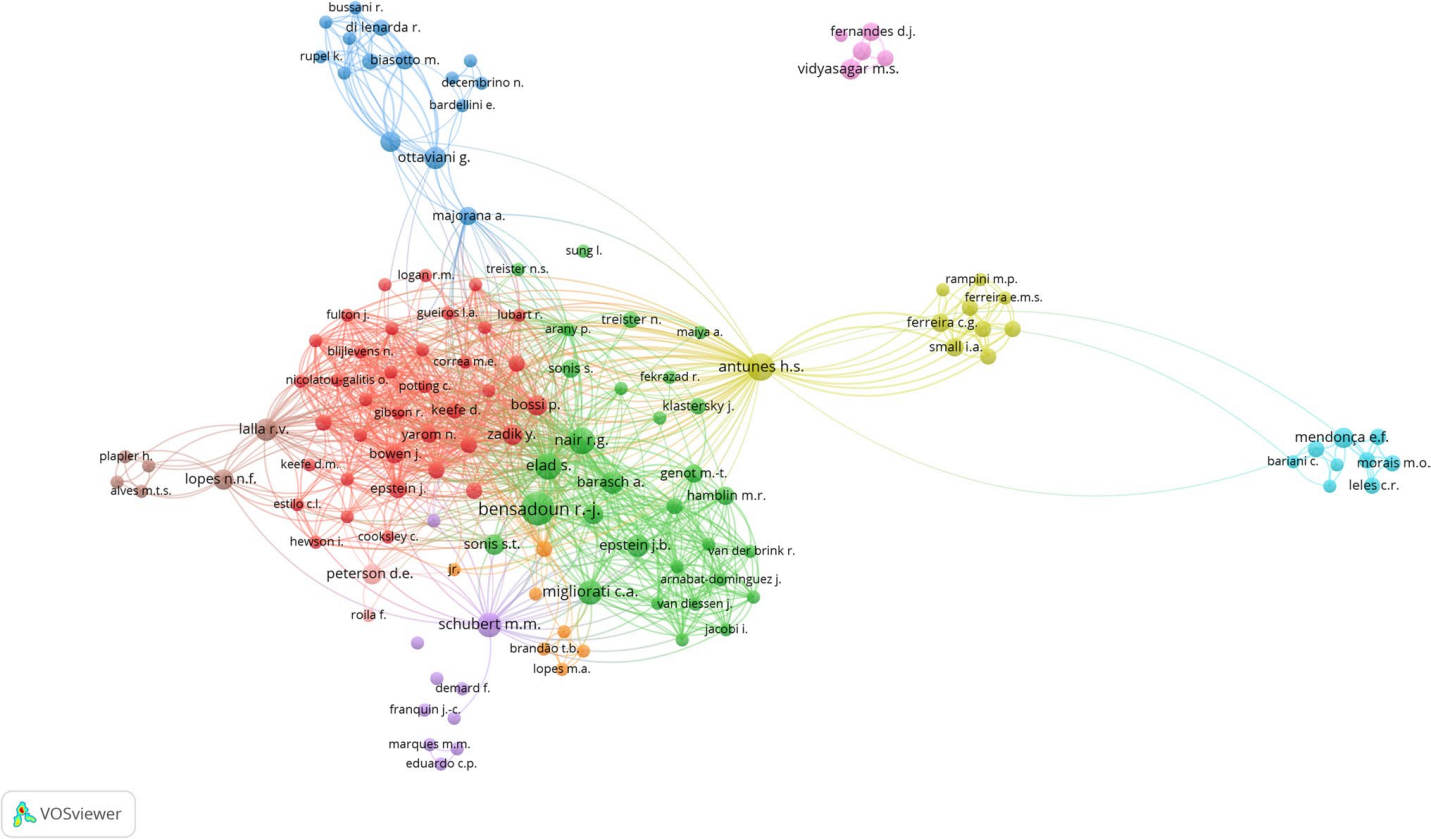
Co-occurrence network of authorships and co-authorships of the most cited articles

In total, 610 individuals, including authors and co-authors, were involved in the 100 articles included in this review. The article with the most collaborators had 90 authors and was also the most cited, with 868 citations. The articles had an average of 9 authors, and 3 articles were authored individually, without collaboration. Upon evaluating the references of the 100 most cited studies, names such as Bensadoun RJ, Schubert MM, Antunes HS, Lalla RV, Elad S, and Epstein JB appeared frequently, indicating that they were highly cited in the references of the articles in this review. The network of authorships and co-authorships can be seen in Fig. 3. The larger the colored circle, the more frequently the author was cited in the references.

The 10 most productive authors and the number of documents in which they appear among the articles included in this analysis are presented in Table 2, along with other related bibliometric indices. The most productive author was Bensadoun RJ, with 14 articles, followed by Antunes HS, Elad S, and Nair RG, each with 8 articles among the 100 most cited. It was observed that 60% of the most productive authors have an H-index greater than 40.

**Table 2:** Authors with the highest number of articles among the most cited and related bibliometric indices

Rank	Author	Started	Citations	Documents	G-index^1^	H-index^2^	M-index^3^
1	Bensadoun RJ	1996	10,473	14	97	50	1.72
2	Antunes HS	2007	1639	8	40	15	0.83
3	Elad S	1996	8585	8	89	51	1.76
4	Nair RG	1995	4280	8	57	23	0.77
5	Migliorati CA	1996	9645	7	97	43	1.48
6	Barasch A	1996	4870	6	69	33	1.14
7	Epstein JB	1980	28,971	6	146	86	1.91
8	Lalla RV	2001	8502	6	91	52	2.17
9	Ottaviani G	2012	1636	6	39	19	1.46
10	Bossi P	2003	11,749	5	95	55	2.50

### Country and filliations production

Brazil was the country with the highest number of articles included in this analysis, with 41 studies, followed by the United States of America (USA) with 34. However, the USA leads the ranking as the country with the highest number of citations. The distribution of articles by country is shown as online resource 3, and the ranking of the ten countries with the highest number of citations is presented in Table 3.

**Table 3:** Ranking of countries with the highest number of citations and article distribution

Rank	Country	Total citation	Average citation	Articles by country
1	USA	2773	198.1	34
2	Brazil	2230	69.7	41
3	France	1230	111.8	18
4	Australia	782	260.7	13
5	Italy	576	82.3	15
6	India	535	89.2	8
7	UK	495	123.8	7
8	Belgium	296	59.2	8
9	Norway	284	142.0	3
10	Israel	255	255	4

**Fig. 4:**
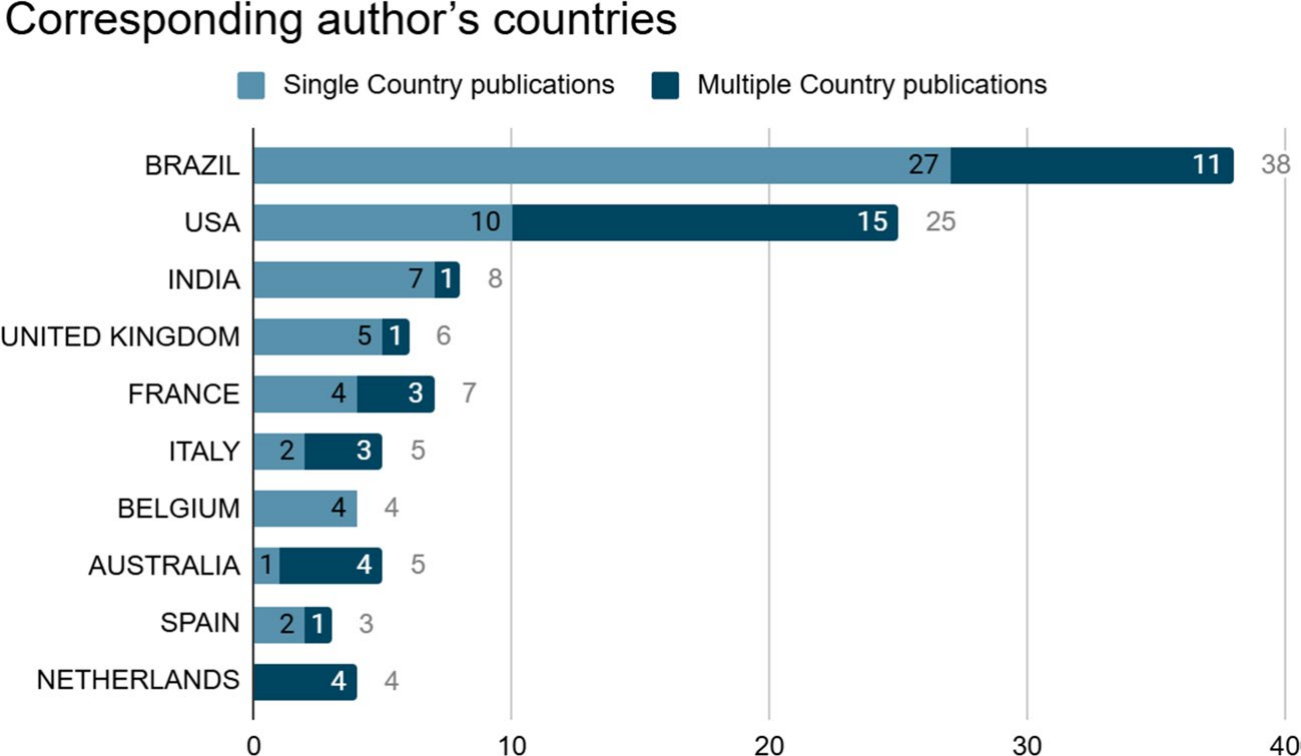
Corresponding author’s countries

It is noteworthy that part of this scientific output involved collaborations between authors from different countries, as illustrated in Fig. 4. The sample with 32 studies showing international collaboration and 68 featuring national or single-country collaboration. The internationally collaborative articles showed an average of 167.4 ± 177.5 citations, and the median was 104 citations. In contrast, studies with national collaboration demonstrated an average of 79.3 ± 42.7 citations per article, and the median was 63 citations (ranging from 43 to 241).

Among the institutions with the highest number of publications were the University of São Paulo (*n* = 9) and the National Cancer Institute (INCA) (*n* = 8), both located in Brazil. The Centre Antoine-Lacassagne (France), Harvard School of Dental Medicine (USA), Manipal University (India), and University of Rochester Medical Center (USA) each had 7 articles. They were followed by the Federal University of Goiás (Brazil), Kaohsiung Medical University (China), and University of Trieste (Italy), each with 6 articles.

### Journals

The 100 most cited articles were published in 52 distinct scientific journals. The journal with the most articles published is *Supportive Care in Cancer*, with 14 studies, followed by *Oral Oncology* with 8, *Laser in Medical Science* with 5, *Photomedicine and Laser Surgery*, and *Cancer* journals, both with 4 articles, as shown in Table 4, with impact factor and average of citations.

**Table 4:** List of journals with the highest number of articles among the 100 most cited

Journal	Articles	Impact factor*	Average citations
*Supportive Care in Cancer*	14	2.8	5.7
*Oral Oncology*	08	4.0	8.7
*Lasers in Medical Science*	05	2.1	5.1
*Photomedicine and Laser Surgery*	04	1.8	4.1
*Cancer*	04	6.1	13.1

*Impact factor 2023

## Discussion

To date, no bibliometric analysis has been published specifically investigating the use of photobiomodulation in oral mucositis. This study, therefore, represents the first to evaluate the characteristics of the 100 most cited articles on this topic. A total of 725 articles were initially identified and screened, focusing exclusively on the use of photobiomodulation for the prevention and treatment of oral mucositis induced by antineoplastic therapies such as chemotherapy and radiotherapy. From this corpus, the 100 most cited studies were selected for in-depth bibliometric analysis. This review enabled the synthesis of key metrics related to authorship, citation performance, countries of origin, and journal distribution, as well as the interactions among these variables.

The bibliometric review is a methodological approach that offers both quantitative and qualitative insights into a given subject and has been increasingly employed to comprehensively map scientific evidence. This strategy holds the potential to generate new research directions by identifying gaps in knowledge and analyzing trends, thereby addressing questions that remain unresolved or only partially answered despite the volume of existing studies [10, 11].

The research strategy was carefully developed to retrieve comprehensive and relevant literature addressing the application of photobiomodulation in the context of oral mucositis. The most cited articles were published between 1993 and 2022, with a notable increase in interest over time, peaking in 2016, which featured the highest concentration of highly cited studies. The vast majority of these publications addressed either the treatment, prevention, or both aspects of oral mucositis in patients undergoing radiotherapy or chemotherapy. This focus is justified by the clinical burden of oral mucositis, which can lead to secondary infections, feeding difficulties, increased reliance on analgesics, and, in severe cases, treatment interruptions, ultimately compromising patient quality of life and contributing to poorer clinical outcomes [12].

Moreover, the global relevance of this topic is reflected in the international collaboration patterns observed in this review. Countries such as Brazil, the USA, France, Australia, Belgium, and India, spanning North America, South America, Europe, Oceania, and Asia, were prominently represented, indicating a widespread and multicontinental research interest in the application of photobiomodulation for oral mucositis management.

Over time, several bibliometric indicators have been developed to quantitatively and qualitatively evaluate scientific journals, offering a means to identify those that consistently publish high-impact research. While citation count alone does not fully capture the impact of a study—given the numerous factors influencing citation behavior—the use of citation-based metrics, particularly in combination, remains one of the most accepted approaches for assessing the academic influence of scientific articles [13].

In this study, the bibliometric indices of the top 10 most cited authors were analyzed, including the H-index, G-index, and M-index. The H-index is commonly used to assess overall researcher impact, while the G-index complements it by emphasizing the influence of highly cited publications, and the M-index accounts for the length of an author’s academic career [14].

Based on these metrics, Bensadoun RJ had the highest number of publications among the most cited articles, while the remaining authors exhibited relatively balanced publication counts. Epstein JB had the highest H-index (86), followed by Bensadoun RJ, Elad S, Migliorati CA, Lalla RV, and Bossi P, all of whom also demonstrated high H-indices, indicating substantial citation impact across their body of work. Epstein JB also registered the highest G-index (146), suggesting significant visibility of his publications. It is important to note that both the H-index and G-index are influenced by the total number of publications. In particular, the G-index tends to favor prolific authors, provided their publications receive at least moderate citation levels [14].

Given that cancer remains a global public health challenge, it is essential for healthcare professionals to understand its complexity to ensure effective prevention, diagnosis, and management, while keeping pace with the continuous evolution of knowledge required for cancer care [15]. Among the most cited articles, seven were identified as clinical guidelines. Notably, the top-ranked article, with 868 citations, was the MASCC/ISOO Clinical Practice Guidelines for the Management of Mucositis Secondary to Cancer Therapy [16]. This publication established evidence-based recommendations for the management of oral mucositis, offering clinicians a structured set of interventions to inform practice and guide decision-making.

Despite the strong evidence supporting the use of photobiomodulation in the prevention and treatment of oral mucositis, certain aspects remain unclear. This review identified ongoing gaps in the literature, particularly regarding the mechanisms of inflammation modulation, tissue repair, and the potential effects of laser therapy on cancer cells. Some of the most cited studies in this domain, published within the past decade, have begun to address these questions.

Brazil stands out as a leading contributor to the literature on photobiomodulation, with the highest number of articles among the top 100 most cited. This highlights the scientific potential of Brazilian researchers and institutions, including the University of São Paulo, the National Cancer Institute (INCA), and the Department of Oral Medicine at the Federal University of Goiás.

According to the criteria proposed by Gehanno et al. [17], which define classic articles as those with more than 100 citations, 31% of the articles included in this bibliometric review may be considered classics, although this threshold is not universally adopted. While frequently debated, citation count remains a valuable metric for understanding the historical trajectory of scientific progress and gauging the influence of research on authors, institutions, and countries [17].

Regarding study design, clinical trials predominated over observational studies. This pattern suggests a strong focus on generating evidence under controlled conditions to evaluate the efficacy and applicability of photobiomodulation in the management of mucositis. Additionally, findings from this review indicate a growing number of secondary studies in the form of systematic reviews. These reviews represent a rigorous method of synthesizing evidence, as they aggregate data from clinical trials that address similar research questions or utilize comparable methodologies [18]. By consolidating evidence, systematic reviews not only provide a comprehensive understanding of a given field but also help identify areas of consensus and highlight gaps in the existing literature.

The journal with the highest number of articles among the 100 most cited is *Supportive Care in Cancer*, published by Springer Science in collaboration with the *Multinational Association of Supportive Care in Cancer* (MASCC). This journal offers current insights into clinical and scientific practices across all aspects of supportive care for cancer patients and reported an impact factor of 2.8 in 2023. The impact factor is a widely used bibliometric indicator that reflects the average number of citations received per paper published in a journal over the preceding 2 years. It is calculated by dividing the number of citations in the current year by the number of citable items published in the previous 2 years.

Among the cited journals, *Cancer* also stands out as a prominent interdisciplinary and international journal publishing high-quality original research, clinical trials, reviews, editorials, and commentaries. It covers topics ranging from etiopathogenesis and epidemiology to prevention and clinical characteristics and currently holds an impact factor of 6.1.

Although bibliometric reviews are primarily quantitative in nature reducing the selection bias often associated with systematic reviews [19], however, this study is subject to certain limitations. One notable limitation is temporal bias, as the analysis required a minimum of 100 citations for article inclusion. This criterion may have favored older publications over more recent ones that have not yet accumulated sufficient citations to meet the inclusion threshold. Additionally, due to the quantitative scope of the bibliometric approach, the included studies were not evaluated for their methodological quality or level of evidence.

## Conclusion

This study represents the first bibliometric analysis to characterize the 100 most cited articles on the use of photobiomodulation for the prevention and treatment of oral mucositis. It provides a comprehensive overview of the scientific output in this field, with an emphasis on studies primarily focused on the clinical efficacy of laser therapy. The set of most cited articles identified herein serves as a valuable reference for both researchers and clinicians, offering insights into the most influential publications, key contributors, high-impact journals, and leading countries in this domain. Furthermore, this bibliometric mapping offers a strategic foundation for identifying emerging themes and knowledge gaps, guiding future research efforts and promoting the development of new investigative pathways in supportive cancer care.

## Supplementary Information

Below is the link to the electronic supplementary material.

ESM 1 (DOCX 28.7 KB)

ESM 2 (TIFF 17.5 KB)

ESM 3 (TIFF 23.7 KB)

## Data Availability

No datasets were generated or analysed during the current study.
