# Supplementary material for: Photobiomodulation therapy in oral mucositis prevention and treatment: insights from a bibliometric review
Source: Support Care Cancer. 2026 Jul 16;34(8):768. doi: 10.1007/s00520-026-10976-5 (PMC13375819; doi:10.1007/s00520-026-10976-5)
Supplement: Supplementary file 1 — (DOCX 28.7 KB) [file 520_2026_10976_MOESM1_ESM.docx]

**Online resource 1 -** List of the 100 articles with the highest number of citations

| **N** | **FIRST AUTHOR** | **LAST AUTHOR** | **TITLE** | **JOURNAL** | **YEAR** | **CITATIONS** |
| --- | --- | --- | --- | --- | --- | --- |
| 1 | LALLA RV | ZADIK Y | MASCC/ISOO CLINICAL PRACTICE GUIDELINES FOR THE MANAGEMENT OF MUCOSITIS SECONDARY TO CANCER THERAPY | CANCER | 2014 | 868 |
| 2 | RUBENSTEIN EB | SONIS ST | CLINICAL PRACTICE GUIDELINES FOR THE PREVENTION AND TREATMENT OF CANCER THERAPY-INDUCED ORAL AND GASTROINTESTINAL MUCOSITIS | CANCER | 2004 | 656 |
| 3 | ELAD S | ZUR E | MASCC/ISOO CLINICAL PRACTICE GUIDELINES FOR THE MANAGEMENT OF MUCOSITIS SECONDARY TO CANCER THERAPY | CANCER | 2020 | 413 |
| 4 | EPSTEIN JB | MAGHAMI E | ORAL COMPLICATIONS OF CANCER AND CANCER THERAPY: FROM CANCER TREATMENT TO SURVIVORSHIP | CA CANCER JOURNAL FOR CLINICIANS | 2012 | 393 |
| 5 | ZADIK Y | ELAD S | SYSTEMATIC REVIEW OF PHOTOBIOMODULATION FOR THE MANAGEMENT OF ORAL MUCOSITIS IN CANCER PATIENTS AND CLINICAL PRACTICE GUIDELINES | SUPPORTIVE CARE IN CANCER | 2019 | 255 |
| 6 | PULITO C | STRANO S | ORAL MUCOSITIS: THE HIDDEN SIDE OF CANCER THERAPY | JOURNAL OF EXPERIMENTAL AND CLINICAL CANCER RESEARCH | 2020 | 241 |
| 7 | VILLA A | SONIS ST | MUCOSITIS: PATHOBIOLOGY AND MANAGEMENT | CURRENT OPINION IN ONCOLOGY | 2015 | 222 |
| 8 | BJORDAL JM | LOPES-MARTINS RA | A SYSTEMATIC REVIEW WITH META-ANALYSIS OF THE EFFECT OF LOW-LEVEL LASER THERAPY (LLLT) IN CANCER THERAPY-INDUCED ORAL MUCOSITIS | SUPPORTIVE CARE IN CANCER | 2011 | 220 |
| 9 | BENSADOUN RJ | DEMARD F | LOW-ENERGY HE/NE LASER IN THE PREVENTION OF RADIATION-INDUCED MUCOSITIS: A MULTICENTER PHASE III RANDOMIZED STUDY IN PATIENTS WITH HEAD AND NECK CANCER | SUPPORTIVE CARE IN CANCER | 1999 | 218 |
| 10 | PETERSON DE | ROILA F | MANAGEMENT OF ORAL AND GASTROINTESTINAL MUCOSITIS: ESMO CLINICAL PRACTICE GUIDELINES | ANNALS OF ONCOLOGY | 2011 | 207 |
| 11 | DUNCAN M | GRANT G | REVIEW ARTICLE: ORAL AND INTESTINAL MUCOSITIS - CAUSES AND POSSIBLE TREATMENTS | ALIMENTARY PHARMACOLOGY AND THERAPEUTICS | 2003 | 202 |
| 12 | COWEN D | FRANQUIN JC | LOW ENERGY HELIUM-NEON LASER IN THE PREVENTION OF ORAL MUCOSITIS IN PATIENTS UNDERGOING BONE MARROW TRANSPLANT: RESULTS OF A DOUBLE BLIND RANDOMIZED TRIAL | INTERNATIONAL JOURNAL OF RADIATION ONCOLOGY BIOLOGY PHYSICS | 1997 | 199 |
| 13 | WORTHINGTON HV | KHALID T | INTERVENTIONS FOR PREVENTING ORAL MUCOSITIS FOR PATIENTS WITH CANCER RECEIVING TREATMENT. | COCHRANE DATABASE OF SYSTEMATIC REVIEWS (ONLINE) | 2010 | 194 |
| 14 | MIGLIORATI C | ELAD | SYSTEMATIC REVIEW OF LASER AND OTHER LIGHT THERAPY FOR THE MANAGEMENT OF ORAL MUCOSITIS IN CANCER PATIENTS | SUPPORTIVE CARE IN CANCER | 2013 | 191 |
| 15 | SCHUBERT MM | HAMDI M | A PHASE III RANDOMIZED DOUBLE-BLIND PLACEBO-CONTROLLED CLINICAL TRIAL TO DETERMINE THE EFFICACY OF LOW LEVEL LASER THERAPY FOR THE PREVENTION OF ORAL MUCOSITIS IN PATIENTS UNDERGOING HEMATOPOIETIC CELL TRANSPLANTATION | SUPPORTIVE CARE IN CANCER | 2007 | 187 |
| 16 | ZECHA JAEM | BENSADOUN RJ | LOW-LEVEL LASER THERAPY/PHOTOBIOMODULATION IN THE MANAGEMENT OF SIDE EFFECTS OF CHEMORADIATION THERAPY IN HEAD AND NECK CANCER: PART 2: PROPOSED APPLICATIONS AND TREATMENT PROTOCOLS | SUPPORTIVE CARE IN CANCER | 2016 | 183 |
| 17 | ZECHA JAEM | BENSADOUN RJ | LOW LEVEL LASER THERAPY/PHOTOBIOMODULATION IN THE MANAGEMENT OF SIDE EFFECTS OF CHEMORADIATION THERAPY IN HEAD AND NECK CANCER: PART 1: MECHANISMS OF ACTION, DOSIMETRIC, AND SAFETY CONSIDERATIONS | SUPPORTIVE CARE IN CANCER | 2016 | 181 |
| 18 | SCULLY C | SONIS S | ORAL MUCOSITIS: A CHALLENGING COMPLICATION OF RADIOTHERAPY, CHEMOTHERAPY, AND RADIOCHEMOTHERAPY: PART 1, PATHOGENESIS AND PROPHYLAXIS OF MUCOSITIS | HEAD AND NECK | 2003 | 175 |
| 19 | CLARKSON JE | MEYER S | INTERVENTIONS FOR TREATING ORAL MUCOSITIS FOR PATIENTS WITH CANCER RECEIVING TREATMENT. | COCHRANE DATABASE OF SYSTEMATIC REVIEWS (ONLINE) | 2010 | 143 |
| 20 | RODRÍGUEZ-CA | GUTIÉRREZ-PÉREZ JL | CANCER TREATMENT-INDUCED ORAL MUCOSITIS: A CRITICAL REVIEW | INTERNATIONAL JOURNAL OF ORAL AND MAXILLOFACIAL SURGERY | 2012 | 142 |
| 21 | ROSENTHAL DI | TROTTI A | STRATEGIES FOR MANAGING RADIATION-INDUCED MUCOSITIS IN HEAD AND NECK CANCER | SEMINARS IN RADIATION ONCOLOGY | 2009 | 142 |
| 22 | OBEROI S | SUNG L | EFFECT OF PROPHYLACTIC LOW LEVEL LASER THERAPY ON ORAL MUCOSITIS: A SYSTEMATIC REVIEW AND META-ANALYSIS | PLOS ONE | 2014 | 133 |
| 23 | MALLICK S | RATH GK | RADIATION INDUCED ORAL MUCOSITIS: A REVIEW OF CURRENT LITERATURE ON PREVENTION AND MANAGEMENT | EUROPEAN ARCHIVES OF OTO-RHINO-LARYNGOLOGY | 2016 | 124 |
| 24 | BARASCH A | TUTSCHKA P | HELIUM‐NEON LASER EFFECTS ON CONDITIONING‐INDUCED ORAL MUCOSITIS IN BONE MARROW TRANSPLANTATION PATIENTS | CANCER | 1995 | 123 |
| 25 | ANTUNES HS | FERREIRA CG | LOW-POWER LASER IN THE PREVENTION OF INDUCED ORAL MUCOSITIS IN BONE MARROW TRANSPLANTATION PATIENTS: A RANDOMIZED TRIAL | BLOOD | 2007 | 116 |
| 26 | BENSADOUN RJ | NAIR RG | LOW-LEVEL LASER THERAPY IN THE PREVENTION AND TREATMENT OF CANCER THERAPY-INDUCED MUCOSITIS: 2012 STATE OF THE ART BASED ON LITERATURE REVIEW AND META-ANALYSIS | CURRENT OPINION IN ONCOLOGY | 2012 | 110 |
| 27 | PLEVOVÁ P | - | PREVENTION AND TREATMENT OF CHEMOTHERAPY- AND RADIOTHERAPY-INDUCED ORAL MUCOSITIS: A REVIEW | ORAL ONCOLOGY | 1999 | 110 |
| 28 | TREISTER N | SONIS S | MUCOSITIS: BIOLOGY AND MANAGEMENT | CURRENT OPINION IN OTOLARYNGOLOGY AND HEAD AND NECK SURGERY | 2007 | 109 |
| 29 | ANTUNES HS | FERREIRA CG | PHASE III TRIAL OF LOW-LEVEL LASER THERAPY TO PREVENT ORAL MUCOSITIS IN HEAD AND NECK CANCER PATIENTS TREATED WITH CONCURRENT CHEMORADIATION | RADIOTHERAPY AND ONCOLOGY | 2013 | 104 |
| 30 | ARORA H | RAJEEV A | EFFICACY OF HE-NE LASER IN THE PREVENTION AND TREATMENT OF RADIOTHERAPY-INDUCED ORAL MUCOSITIS IN ORAL CANCER PATIENTS | ORAL SURGERY, ORAL MEDICINE, ORAL PATHOLOGY, ORAL RADIOLOGY AND ENDODONTOLOGY | 2008 | 103 |
| 31 | KUHN A | BRUNETTO AL | LOW-LEVEL INFRARED LASER THERAPY IN CHEMOTHERAPY-INDUCED ORAL MUCOSITIS: A RANDOMIZED PLACEBO-CONTROLLED TRIAL IN CHILDREN | JOURNAL OF PEDIATRIC HEMATOLOGY/ONCOLOGY | 2009 | 101 |
| 32 | SONIS ST | RABER-DURLACHER JE | COULD THE BIOLOGICAL ROBUSTNESS OF LOW LEVEL LASER THERAPY (PHOTOBIOMODULATION) IMPACT ITS USE IN THE MANAGEMENT OF MUCOSITIS IN HEAD AND NECK CANCER PATIENTS | ORAL ONCOLOGY | 2016 | 97 |
| 33 | SIMÕES A | EDUARDO CP | LASER PHOTOTHERAPY AS TOPICAL PROPHYLAXIS AGAINST HEAD AND NECK CANCER RADIOTHERAPY-INDUCED ORAL MUCOSITIS: COMPARISON BETWEEN LOW AND HIGH/LOW POWER LASERS | LASERS IN SURGERY AND MEDICINE | 2009 | 95 |
| 34 | ANTUNES HS | FERREIRA CG | LONG-TERM SURVIVAL OF A RANDOMIZED PHASE III TRIAL OF HEAD AND NECK CANCER PATIENTS RECEIVING CONCURRENT CHEMORADIATION THERAPY WITH OR WITHOUT LOW-LEVEL LASER THERAPY (LLLT) TO PREVENT ORAL MUCOSITIS | ORAL ONCOLOGY | 2017 | 92 |
| 35 | DE LIMA AG | SNITCOVSKY IML | ORAL MUCOSITIS PREVENTION BY LOW-LEVEL LASER THERAPY IN HEAD-AND-NECK CANCER PATIENTS UNDERGOING CONCURRENT CHEMORADIOTHERAPY: A PHASE III RANDOMIZED STUDY | INTERNATIONAL JOURNAL OF RADIATION ONCOLOGY BIOLOGY PHYSICS | 2012 | 90 |
| 36 | CARVALHO PAG | ALVES FA | EVALUATION OF LOW-LEVEL LASER THERAPY IN THE PREVENTION AND TREATMENT OF RADIATION-INDUCED MUCOSITIS: A DOUBLE-BLIND RANDOMIZED STUDY IN HEAD AND NECK CANCER PATIENTS | ORAL ONCOLOGY | 2011 | 89 |
| 37 | CRUZ LB | BRUNETTO AL | INFLUENCE OF LOW-ENERGY LASER IN THE PREVENTION OF ORAL MUCOSITIS IN CHILDREN WITH CANCER RECEIVING CHEMOTHERAPY | PEDIATRIC BLOOD AND CANCER | 2007 | 89 |
| 38 | GAUTAM AP | NIGUDGI S | EFFECT OF LOW-LEVEL LASER THERAPY ON PATIENT REPORTED MEASURES OF ORAL MUCOSITIS AND QUALITY OF LIFE IN HEAD AND NECK CANCER PATIENTS RECEIVING CHEMORADIOTHERAPY - A RANDOMIZED CONTROLLED TRIAL | SUPPORTIVE CARE IN CANCER | 2013 | 88 |
| 39 | MAIYA GA | FERNANDES D | EFFECT OF LOW LEVEL HELIUM-NEON (HE-NE) LASER THERAPY IN THE PREVENTION & TREATMENT OF RADIATION INDUCED MUCOSITIS IN HEAD & NECK CANCER PATIENTS | INDIAN JOURNAL OF MEDICAL RESEARCH | 2006 | 88 |
| 40 | HE M | SUN J | A SYSTEMATIC REVIEW AND META-ANALYSIS OF THE EFFECT OF LOW-LEVEL LASER THERAPY (LLLT) ON CHEMOTHERAPY-INDUCED ORAL MUCOSITIS IN PEDIATRIC AND YOUNG PATIENTS | EUROPEAN JOURNAL OF PEDIATRICS | 2018 | 87 |
| 41 | GAUTAM AP | VADHIRAJA BM | LOW LEVEL LASER THERAPY FOR CONCURRENT CHEMORADIOTHERAPY INDUCED ORAL MUCOSITIS IN HEAD AND NECK CANCER PATIENTS - A TRIPLE BLINDED RANDOMIZED CONTROLLED TRIAL | RADIOTHERAPY AND ONCOLOGY | 2012 | 85 |
| 42 | ABRAMOFF MMF | PETRILLI AS | LOW-LEVEL LASER THERAPY IN THE PREVENTION AND TREATMENT OF CHEMOTHERAPY-INDUCED ORAL MUCOSITIS IN YOUNG PATIENTS | PHOTOMEDICINE AND LASER SURGERY | 2008 | 85 |
| 43 | SPERANDIO FF | DE SOUSA SCOM | LOW-LEVEL LASER THERAPY CAN PRODUCE INCREASED AGGRESSIVENESS OF DYSPLASTIC AND ORAL CANCER CELL LINES BY MODULATION OF AKT/MTOR SIGNALING PATHWAY | JOURNAL OF BIOPHOTONICS | 2013 | 84 |
| 44 | OTON-LEITE AF | MENDONÇA EF | EFFECT OF LOW-LEVEL LASER THERAPY ON CHEMORADIOTHERAPY-INDUCED ORAL MUCOSITIS AND SALIVARY INFLAMMATORY MEDIATORS IN HEAD AND NECK CANCER PATIENTS | LASERS IN SURGERY AND MEDICINE | 2015 | 83 |
| 45 | LOPES NNF | ALVES MTS | CYCLOOXYGENASE-2 AND VASCULAR ENDOTHELIAL GROWTH FACTOR EXPRESSION IN 5-FLUOROURACIL-INDUCED ORAL MUCOSITIS IN HAMSTERS: EVALUATION OF TWO LOW-INTENSITY LASER PROTOCOLS | SUPPORTIVE CARE IN CANCER | 2009 | 83 |
| 46 | ROBIJNS J | BENSADOUN RJ | PHOTOBIOMODULATION THERAPY IN MANAGEMENT OF CANCER THERAPY-INDUCED SIDE EFFECTS: WALT POSITION PAPER 2022 | FRONTIERS IN ONCOLOGY | 2022 | 79 |
| 47 | DE PAULI PAGLIONI M | SANTOS-SILVA AR | TUMOR SAFETY AND SIDE EFFECTS OF PHOTOBIOMODULATION THERAPY USED FOR PREVENTION AND MANAGEMENT OF CANCER TREATMENT TOXICITIES. A SYSTEMATIC REVIEW | ORAL ONCOLOGY | 2019 | 79 |
| 48 | SILVA GBL | SILVA MAG | THE PREVENTION OF INDUCED ORAL MUCOSITIS WITH LOW-LEVEL LASER THERAPY IN BONE MARROW TRANSPLANTATION PATIENTS: A RANDOMIZED CLINICAL TRIAL | PHOTOMEDICINE AND LASER SURGERY | 2011 | 73 |
| 49 | KUSIAK A | ALTERIO D | ONCOLOGICAL-THERAPY RELATED ORAL MUCOSITIS AS AN INTERDISCIPLINARY PROBLEM—LITERATURE REVIEW | INTERNATIONAL JOURNAL OF ENVIRONMENTAL RESEARCH AND PUBLIC HEALTH | 2020 | 71 |
| 50 | BASSO FG | HEBLING J | PROLIFERATION, MIGRATION, AND EXPRESSION OF ORAL-MUCOSAL-HEALING-RELATED GENES BY ORAL FIBROBLASTS RECEIVING LOW-LEVEL LASER THERAPY AFTER INFLAMMATORY CYTOKINES CHALLENGE | LASERS IN SURGERY AND MEDICINE | 2016 | 69 |
| 51 | VAN SEBILLE YZA | KEEFE DM | MANAGEMENT OF MUCOSITIS DURING CHEMOTHERAPY: FROM PATHOPHYSIOLOGY TO PRAGMATIC THERAPEUTICS | CURRENT ONCOLOGY REPORTS | 2015 | 66 |
| 52 | LOPES NNF | ALVES MTS | EFFECTS OF LOW-LEVEL LASER THERAPY ON COLLAGEN EXPRESSION AND NEUTROPHIL INFILTRATE IN 5-FLUOROURACIL-INDUCED ORAL MUCOSITIS IN HAMSTERS | LASERS IN SURGERY AND MEDICINE | 2010 | 66 |
| 53 | CRONSHAW M | GROOTVELD M | PHOTOBIOMODULATION AND ORAL MUCOSITIS: A SYSTEMATIC REVIEW | DENTISTRY JOURNAL | 2020 | 64 |
| 54 | OTTAVIANI G | ZACCHIGNA S | LASER THERAPY INHIBITS TUMOR GROWTH IN MICE BY PROMOTING IMMUNE SURVEILLANCE AND VESSEL NORMALIZATION | EBIOMEDICINE | 2016 | 64 |
| 55 | D'HONDT L | CANON JL | ORAL MUCOSITIS INDUCED BY ANTICANCER TREATMENTS: PHYSIOPATHOLOGY AND TREATMENTS | THERAPEUTICS AND CLINICAL RISK MANAGEMENT | 2006 | 64 |
| 56 | NES AG | POSSO MBS | PATIENTS WITH MODERATE CHEMOTHERAPY-INDUCED MUCOSITIS: PAIN THERAPY USING LOW INTENSITY LASERS | INTERNATIONAL NURSING REVIEW | 2005 | 64 |
| 57 | BENSADOUN RJ | DEMARD F | CHEMOTHERAPY- AND RADIOTHERAPY-INDUCED MUCOSITIS IN HEAD AND NECK CANCER PATIENTS: NEW TRENDS IN PATHOPHYSIOLOGY, PREVENTION AND TREATMENT | EUROPEAN ARCHIVES OF OTO-RHINO-LARYNGOLOGY | 2001 | 64 |
| 58 | HODGSON BD | WHELAN HT | AMELIORATION OF ORAL MUCOSITIS PAIN BY NASA NEAR-INFRARED LIGHT-EMITTING DIODES IN BONE MARROW TRANSPLANT PATIENTS | SUPPORTIVE CARE IN CANCER | 2012 | 63 |
| 59 | WONG SF | WILDER-SMITH P | PILOT STUDY OF LASER EFFECTS ON ORAL MUCOSITIS IN PATIENTS RECEIVING CHEMOTHERAPY | CANCER JOURNAL | 2002 | 63 |
| 60 | USUMEZ A | GUTKNECHT N | EFFECTS OF LASER IRRADIATION AT DIFFERENT WAVELENGTHS (660, 810, 980, AND 1,064 NM) ON MUCOSITIS IN AN ANIMAL MODEL OF WOUND HEALING | LASERS IN MEDICAL SCIENCE | 2014 | 62 |
| 61 | OTON-LEITE AF | MENDONÇA EF | EFFECT OF LOW LEVEL LASER THERAPY IN THE REDUCTION OF ORAL COMPLICATIONS IN PATIENTS WITH CANCER OF THE HEAD AND NECK SUBMITTED TO RADIOTHERAPY | SPECIAL CARE IN DENTISTRY | 2013 | 62 |
| 62 | CAUWELS RGEC | MARTENS LC | LOW LEVEL LASER THERAPY IN ORAL MUCOSITIS: A PILOT STUDY | EUROPEAN ARCHIVES OF PAEDIATRIC DENTISTRY | 2011 | 62 |
| 63 | VERDI CJ | - | CANCER THERAPY AND ORAL MUCOSITIS: AN APPRAISAL OF DRUG PROPHYLAXIS | DRUG SAFETY | 1993 | 61 |
| 64 | QUTOB AF | KEEFE D | PREVENTION OF ORAL MUCOSITIS IN CHILDREN RECEIVING CANCER THERAPY: A SYSTEMATIC REVIEW AND EVIDENCE-BASED ANALYSIS | ORAL ONCOLOGY | 2013 | 60 |
| 65 | ZANIN T | BRUGNERA A | USE OF 660-NM DIODE LASER IN THE PREVENTION AND TREATMENT OF HUMAN ORAL MUCOSITIS INDUCED BY RADIOTHERAPY AND CHEMOTHERAPY | PHOTOMEDICINE AND LASER SURGERY | 2010 | 60 |
| 66 | BRANDÃO TB | SANTOS-SILVA AR | LOCALLY ADVANCED ORAL SQUAMOUS CELL CARCINOMA PATIENTS TREATED WITH PHOTOBIOMODULATION FOR PREVENTION OF ORAL MUCOSITIS: RETROSPECTIVE OUTCOMES AND SAFETY ANALYSES | SUPPORTIVE CARE IN CANCER | 2018 | 59 |
| 67 | AMADORI F | MAJORANA A | LOW-LEVEL LASER THERAPY FOR TREATMENT OF CHEMOTHERAPY-INDUCED ORAL MUCOSITIS IN CHILDHOOD: A RANDOMIZED DOUBLE-BLIND CONTROLLED STUDY | LASERS IN MEDICAL SCIENCE | 2016 | 59 |
| 68 | GAUTAM AP | GUDDATTU V | LOW LEVEL LASER THERAPY AGAINST RADIATION INDUCED ORAL MUCOSITIS IN ELDERLY HEAD AND NECK CANCER PATIENTS-A RANDOMIZED PLACEBO CONTROLLED TRIAL | JOURNAL OF PHOTOCHEMISTRY AND PHOTOBIOLOGY B: BIOLOGY | 2015 | 59 |
| 69 | GENOT-KLASTERSKY MT | PAESMANS M | THE USE OF LOW-ENERGY LASER (LEL) FOR THE PREVENTION OF CHEMOTHERAPY- AND/OR RADIOTHERAPY-INDUCED ORAL MUCOSITIS IN CANCER PATIENTS: RESULTS FROM TWO PROSPECTIVE STUDIES | SUPPORTIVE CARE IN CANCER | 2008 | 59 |
| 70 | GENOT MT | KLASTERSKY J | LOW-LEVEL LASER FOR PREVENTION AND THERAPY OF ORAL MUCOSITIS INDUCED BY CHEMOTHERAPY OR RADIOTHERAPY | CURRENT OPINION IN ONCOLOGY | 2005 | 59 |
| 71 | GOBBO M | ZANAZZO GA | MULTICENTER RANDOMIZED, DOUBLE-BLIND CONTROLLED TRIAL TO EVALUATE THE EFFICACY OF LASER THERAPY FOR THE TREATMENT OF SEVERE ORAL MUCOSITIS INDUCED BY CHEMOTHERAPY IN CHILDREN: LAMPO RCT | PEDIATRIC BLOOD AND CANCER | 2018 | 58 |
| 72 | RUPEL K | ZACCHIGNA S | PHOTOBIOMODULATION AT MULTIPLE WAVELENGTHS DIFFERENTIALLY MODULATES OXIDATIVE STRESS IN VITRO AND IN VIVO | OXIDATIVE MEDICINE AND CELLULAR LONGEVITY | 2018 | 57 |
| 73 | SILVA GBL | BATISTA AC | EFFECT OF LOW-LEVEL LASER THERAPY ON INFLAMMATORY MEDIATOR RELEASE DURING CHEMOTHERAPY-INDUCED ORAL MUCOSITIS: A RANDOMIZED PRELIMINARY STUDY | LASERS IN MEDICAL SCIENCE | 2015 | 57 |
| 74 | RHEE YH | AHN JC | LOW-LEVEL LASER THERAPY PROMOTED AGGRESSIVE PROLIFERATION AND ANGIOGENESIS THROUGH DECREASING OF TRANSFORMING GROWTH FACTOR-Β1 AND INCREASING OF AKT/HYPOXIA INDUCIBLE FACTOR-1Α IN ANAPLASTIC THYROID CANCER | PHOTOMEDICINE AND LASER SURGERY | 2016 | 56 |
| 75 | CRONSHAW M | GROOTVELD M | PHOTOBIOMODULATION DOSE PARAMETERS IN DENTISTRY: A SYSTEMATIC REVIEW AND META-ANALYSIS | DENTISTRY JOURNAL | 2020 | 54 |
| 76 | BENSADOUN RJ | - | PHOTOBIOMODULATION OR LOW-LEVEL LASER THERAPY IN THE MANAGEMENT OF CANCER THERAPY-INDUCED MUCOSITIS, DERMATITIS AND LYMPHEDEMA | CURRENT OPINION IN ONCOLOGY | 2018 | 54 |
| 77 | CARNEIRO-NETO JN | DE-ANDRADE CR | PROTOCOLS FOR MANAGEMENT OF ORAL COMPLICATIONS OF CHEMOTHERAPY AND/OR RADIOTHERAPY FOR ORAL CANCER: SYSTEMATIC REVIEW AND META-ANALYSIS CURRENT | MEDICINA ORAL PATOLOGIA ORAL Y CIRUGIA BUCAL | 2017 | 54 |
| 78 | BAMPS M | NUYTS S | LOW-LEVEL LASER THERAPY STIMULATES PROLIFERATION IN HEAD AND NECK SQUAMOUS CELL CARCINOMA CELLS | FRONTIERS IN ONCOLOGY | 2018 | 52 |
| 79 | OTON-LEITE AF | MENDONÇA EF | EFFECT OF INTRAORAL LOW-LEVEL LASER THERAPY ON QUALITY OF LIFE OF PATIENTS WITH HEAD AND NECK CANCER UNDERGOING RADIOTHERAPY | HEAD AND NECK | 2012 | 52 |
| 80 | BEZINELLI LM | MICHEL-CROSATO E | COST-EFFECTIVENESS OF THE INTRODUCTION OF SPECIALIZED ORAL CARE WITH LASER THERAPY IN HEMATOPOIETIC STEM CELL TRANSPLANTATION | HEMATOLOGICAL ONCOLOGY | 2014 | 51 |
| 81 | FRANÇA CM | RIBEIRO MS | LOW-INTENSITY RED LASER ON THE PREVENTION AND TREATMENT OF INDUCED-ORAL MUCOSITIS IN HAMSTERS | JOURNAL OF PHOTOCHEMISTRY AND PHOTOBIOLOGY B: BIOLOGY | 2009 | 51 |
| 82 | KHOURI VY | VOLTARELLI JC | USE OF THERAPEUTIC LASER FOR PREVENTION AND TREATMENT OF ORAL MUCOSITIS | BRAZILIAN DENTAL JOURNAL | 2009 | 51 |
| 83 | JAGUAR GC | ALVES FA | LOW-ENERGY LASER THERAPY FOR PREVENTION OF ORAL MUCOSITIS IN HEMATOPOIETIC STEM CELL TRANSPLANTATION | ORAL DISEASES | 2007 | 51 |
| 84 | MIRANDA-SILVA W | ELAD S | MASCC/ISOO CLINICAL PRACTICE GUIDELINES FOR THE MANAGEMENT OF MUCOSITIS: SUB-ANALYSIS OF CURRENT INTERVENTIONS FOR THE MANAGEMENT OF ORAL MUCOSITIS IN PEDIATRIC CANCER PATIENTS | SUPPORTIVE CARE IN CANCER | 2021 | 50 |
| 85 | ANSCHAU F | STEIN AT | EFFICACY OF LOW-LEVEL LASER FOR TREATMENT OF CANCER ORAL MUCOSITIS: A SYSTEMATIC REVIEW AND META-ANALYSIS | LASERS IN MEDICAL SCIENCE | 2019 | 50 |
| 86 | OTTAVIANI G | ZACCHIGNA S | EFFECT OF CLASS IV LASER THERAPY ON CHEMOTHERAPY-INDUCED ORAL MUCOSITIS: A CLINICAL AND EXPERIMENTAL STUDY | AMERICAN JOURNAL OF PATHOLOGY | 2013 | 50 |
| 87 | LEGOUTÉ F | JADAUD E | LOW-LEVEL LASER THERAPY IN TREATMENT OF CHEMORADIOTHERAPY-INDUCED MUCOSITIS IN HEAD AND NECK CANCER: RESULTS OF A RANDOMISED, TRIPLE BLIND, MULTICENTRE PHASE III TRIAL | RADIATION ONCOLOGY | 2019 | 49 |
| 88 | ANTUNES HS | FERREIRA CG | COST-EFFECTIVENESS OF LOW-LEVEL LASER THERAPY (LLLT) IN HEAD AND NECK CANCER PATIENTS RECEIVING CONCURRENT CHEMORADIATION | ORAL ONCOLOGY | 2016 | 48 |
| 89 | VITALE MC | DEFABIANIS P | PRELIMINARY STUDY IN A NEW PROTOCOL FOR THE TREATMENT OF ORAL MUCOSITIS IN PEDIATRIC PATIENTS UNDERGOING HEMATOPOIETIC STEM CELL TRANSPLANTATION (HSCT) AND CHEMOTHERAPY (CT) | LASERS IN MEDICAL SCIENCE | 2017 | 47 |
| 90 | GAUTAM AP | MAIYA GA | LOW LEVEL HELIUM NEON LASER THERAPY FOR CHEMORADIOTHERAPY INDUCED ORAL MUCOSITIS IN ORAL CANCER PATIENTS - A RANDOMIZED CONTROLLED TRIAL | ORAL ONCOLOGY | 2012 | 47 |
| 91 | PETERSON DE | ROILA F | MANAGEMENT OF ORAL AND GASTROINTESTINAL MUCOSITIS: ESMO CLINICAL RECOMMENDATIONS | ANNALS OF ONCOLOGY | 2009 | 47 |
| 92 | DE LIMA AG | VILLAR RC | EFFICACY OF LOW-LEVEL LASER THERAPY AND ALUMINUM HYDROXIDE IN PATIENTS WITH CHEMOTHERAPY AND RADIOTHERAPY-INDUCED ORAL MUCOSITIS | BRAZILIAN DENTAL JOURNAL | 2010 | 46 |
| 93 | BIRON P | BLAY JY | RESEARCH CONTROVERSIES IN MANAGEMENT OF ORAL MUCOSITIS | SUPPORTIVE CARE IN CANCER | 2000 | 46 |
| 94 | MEDEIROS-FILHO JB | FERREIRA MC | LASER AND PHOTOCHEMOTHERAPY FOR THE TREATMENT OF ORAL MUCOSITIS IN YOUNG PATIENTS: RANDOMIZED CLINICAL TRIAL | PHOTODIAGNOSIS AND PHOTODYNAMIC THERAPY | 2017 | 45 |
| 95 | EDUARDO FDP | CORREA L | ORAL MUCOSITIS IN PEDIATRIC PATIENTS UNDERGOING HEMATOPOIETIC STEM CELL TRANSPLANTATION: CLINICAL OUTCOMES IN A CONTEXT OF SPECIALIZED ORAL CARE USING LOW-LEVEL LASER THERAPY | PEDIATRIC TRANSPLANTATION | 2015 | 45 |
| 96 | CHEN CH | HUNG CH | EFFECTS OF LOW-LEVEL LASER THERAPY ON M1-RELATED CYTOKINE EXPRESSION IN MONOCYTES VIA HISTONE MODIFICATION | MEDIATORS OF INFLAMMATION | 2014 | 45 |
| 97 | MARÍN-CONDE F | TORRES-LAGARES D | PHOTOBIOMODULATION WITH LOW-LEVEL LASER THERAPY REDUCES ORAL MUCOSITIS CAUSED BY HEAD AND NECK RADIO-CHEMOTHERAPY: PROSPECTIVE RANDOMIZED CONTROLLED TRIAL | INTERNATIONAL JOURNAL OF ORAL AND MAXILLOFACIAL SURGERY | 2019 | 44 |
| 98 | FEKRAZAD R | CHINIFORUSH N | ORAL MUCOSITIS PREVENTION AND MANAGEMENT BY THERAPEUTIC LASER IN HEAD AND NECK CANCERS | JOURNAL OF LASERS IN MEDICAL SCIENCES | 2014 | 44 |
| 99 | PATEL P | SUNG L | CLINICAL PRACTICE GUIDELINE FOR THE PREVENTION OF ORAL AND OROPHARYNGEAL MUCOSITIS IN PEDIATRIC CANCER AND HEMATOPOIETIC STEM CELL TRANSPLANT PATIENTS: 2021 UPDATE | EUROPEAN JOURNAL OF CANCER | 2021 | 43 |
| 100 | BASSO FG | HEBLING J | BIOMODULATION OF INFLAMMATORY CYTOKINES RELATED TO ORAL MUCOSITIS BY LOW-LEVEL LASER THERAPY | PHOTOCHEMISTRY AND PHOTOBIOLOGY | 2015 | 43 |
